# Challenging the Norm: Occurrence of Synchronous Pleural and Peritoneal Mesothelioma in a Female Patient

**DOI:** 10.7759/cureus.67118

**Published:** 2024-08-18

**Authors:** Surendar Reddy, Kavya M G, Rakesh Gattu, Alekya P, Manasa Kuthadi

**Affiliations:** 1 Pulmonology, ESIC Medical College & Hospital, Hyderabad, IND

**Keywords:** medical thoracoscopy, malignant pleural effusion, pleural interventions, malignant pleural mesothelioma (mpm), peritoneal mesothelioma

## Abstract

Here, we present a unique case involving a female patient in her 40s with synchronous malignant pleural and peritoneal mesothelioma, despite lacking a history of asbestos exposure. The patient's initial symptoms included dyspnoea, chest pain, cough, fever, appetite loss, and weight loss over a month. Clinical evaluation led to the identification of right-sided pleural effusion, prompting consideration of differential diagnoses, such as tubercular or malignant pleural effusion. A thoracoscopy-guided biopsy, followed by histopathological examination and immunohistochemical staining, confirmed the diagnosis of mesothelioma. Chemotherapy was initiated as part of the treatment plan. The prognosis for this condition is generally bad; however, unusual cases of extended survival have been documented. The complexities of our case underscore the critical necessity for a thorough and aggressive evaluation of pleural effusion cases to unveil rare underlying causes, such as mesothelioma.

## Introduction

The incidence of mesothelioma, a rare cancer, was a mere 0.17% of all estimated cancer cases in 2020 [1]. It is widely acknowledged that inhalational exposure to asbestos is the primary cause of malignant mesothelioma in humans [2]. This cancer originates from mesothelial cells of serosal membranes, particularly in the pleura (75%), peritoneum (10-0%), pericardium (1%), or tunica vaginalis (1%) [3]. Concurrent pleural and peritoneal mesothelioma is exceedingly rare [3]. The disease is typically diagnosed in individuals aged between their fifth and seventh decades, with a higher prevalence in men, accounting for approximately 70-80% of cases [3]. This gender disparity is attributed to the greater presence of men in industries with elevated asbestos exposure, such as mining, shipbuilding, and construction [1].

Pleural mesothelioma commonly presents with symptoms like worsening dyspnea, weight loss, and chest wall discomfort, often accompanied by unilateral hemorrhagic pleural effusion and pleural thickening [2]. Conversely, peritoneal mesotheliomas often manifest with abdominal pain, nausea, vomiting, ascites, and accelerated weight loss [3]. Radiographic findings, such as pleural effusion or pleural thickening on plain chest X-rays, necessitate confirmation through contrast-enhanced computed tomography. Although magnetic resonance imaging (MRI) is an option, it typically does not offer significant advantages over CT scans [2]. CT or MRI scans may reveal chest wall invasion, providing additional evidence of malignancy. Fluorodeoxyglucose positron emission tomography (FDG-PET) has proven valuable in diagnosis, particularly in differentiating between benign and malignant conditions [2]. Microscopic differentiation of malignant pleural mesothelioma (MPM) from other tumors, such as adenocarcinoma and other spindle cell tumors, is crucial. Calretinin and WT-1 markers, when combined with other carcinoma markers, are particularly valuable, offering high specificity for MPM [2].

The efficacy of systemic anticancer therapy for MPM has been limited until now although there are growing interest and data supporting the potential of immunotherapy with immune checkpoint inhibition [2]. Between 2011 and 2017, individuals diagnosed with mesothelioma had a five-year relative survival rate of 12%, significantly lower than the 62.7% five-year survival rate observed for all cancers [1]. This article presents a comprehensive case report detailing such an instance in a female, shedding light on the diagnostic challenges encountered in addressing this complex medical condition.

## Case presentation

A middle-aged woman presented with a one-month history of breathing difficulties, chest pain, cough, fever, loss of appetite, and weight loss. She did not have any existing health conditions and had not been exposed to tuberculosis. She was not a smoker or a substance abuser. She lived in a rural area with her husband and two children. There was no asbestos in her living environment.

An initial evaluation revealed an elevated heart rate and normal blood pressure, and her breathing rate was slightly increased. Oxygen levels in her blood were normal. There were no signs of anemia, clubbing, bluish discoloration, jaundice, or significant swelling of lymph nodes. A physical examination indicated a dull note on percussion and reduced breath-sound intensity in specific areas of her chest, suggesting the possibility of right-sided pleural effusion. Despite being initially treated elsewhere and undergoing thoracocentesis and antitubercular therapy due to certain findings in the fluid from her chest, her condition did not improve. Pleural fluid analysis suggested low adenosine deaminase (ADA) and exudative pleural effusion (Table 1).

**Table 1:** Pleural fluid analysis

Pleural fluid parameters	Values	Reference values [4]
Protein	5.48 g/dL	1-2 g/dL
Sugar	148 mg/dL	90-120 mg/dL
Adenosine deaminase	34.3 IU/L	<40 IU/L
Lactate dehydrogenase	673 IU/L	<50% of plasma value
Cytology	Lymphocytes 95%, neutrophils 5%	Lymphocytes 5%, neutrophils 5%
Cellblock	No atypical cells	-

**Figure 1:**
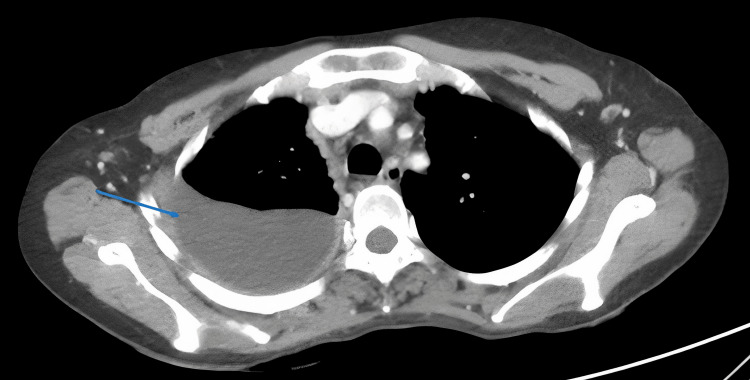
Contrast-enhanced CT scan of the chest showing right-sided pleural effusion (blue arrow).

**Figure 2:**
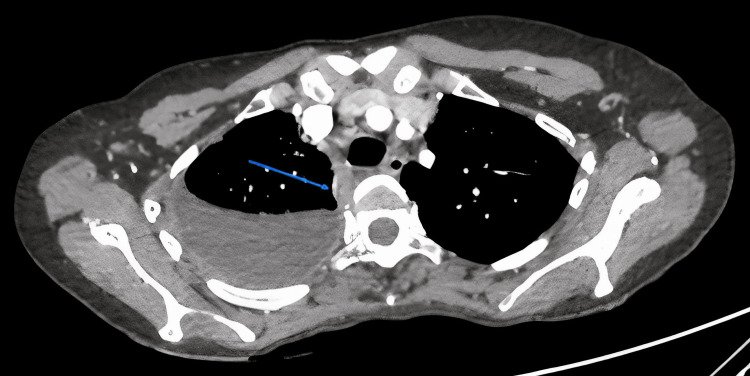
Contrast-enhanced CT scan of the chest showing right-sided pleural effusion with pleural thickening and nodularity (blue arrow).

The contrast-enhanced CT chest imaging demonstrated right-side moderate pleural effusion (Figure 1), thickening of the right pleura with noticeable nodularity, and a maximum thickness of 6 mm (Figure 2).

**Figure 3:**
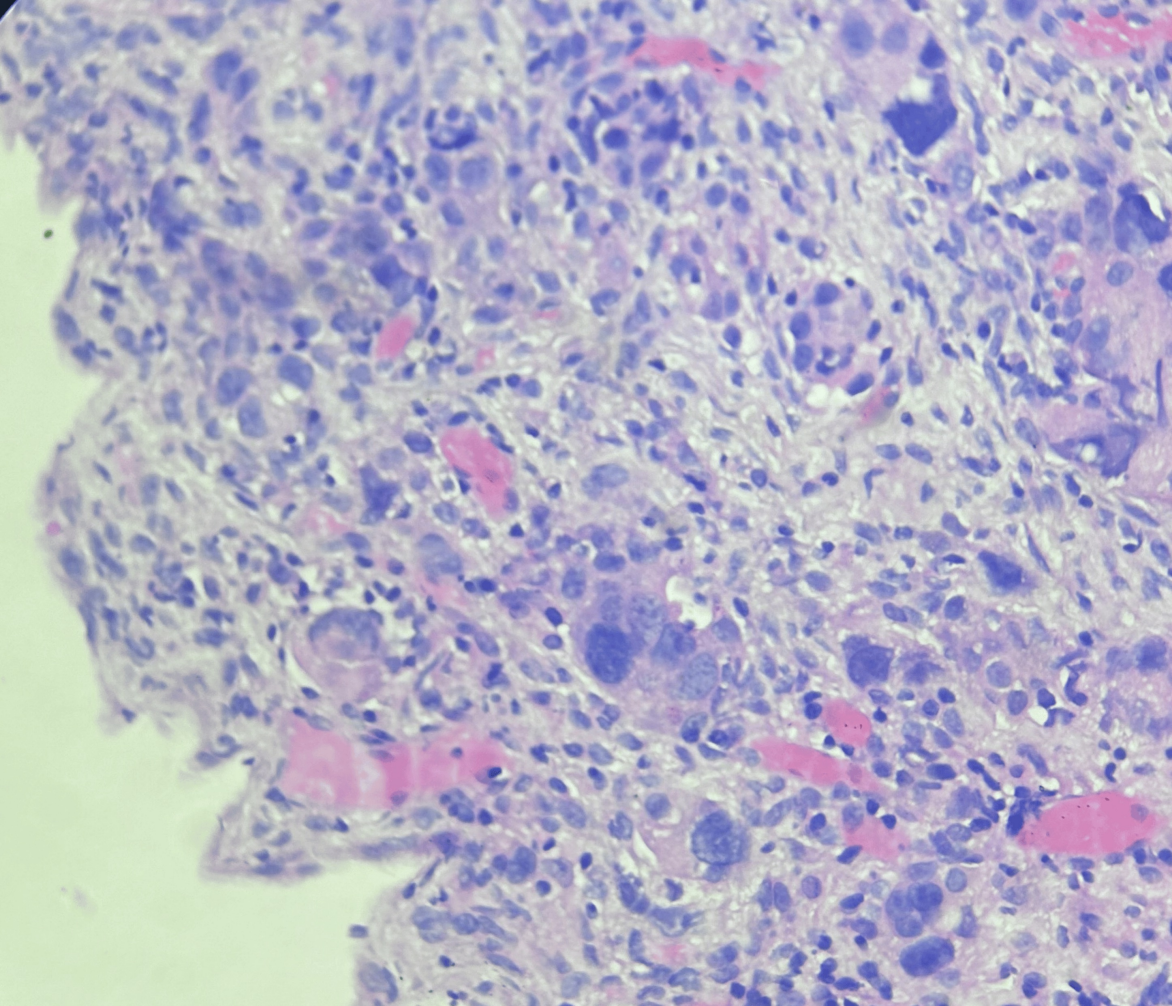
Histopathology image showing atypical cells in parietal pleura (hematoxylin and eosin staining). Magnification 40X

Subsequently, a medical thoracoscopy with pleural biopsy was performed due to heightened concerns about malignancy. Whitish to yellowish nodules were observed during the procedure, and the biopsy was sent for histopathological examination (Figure 3).

**Figure 4:**
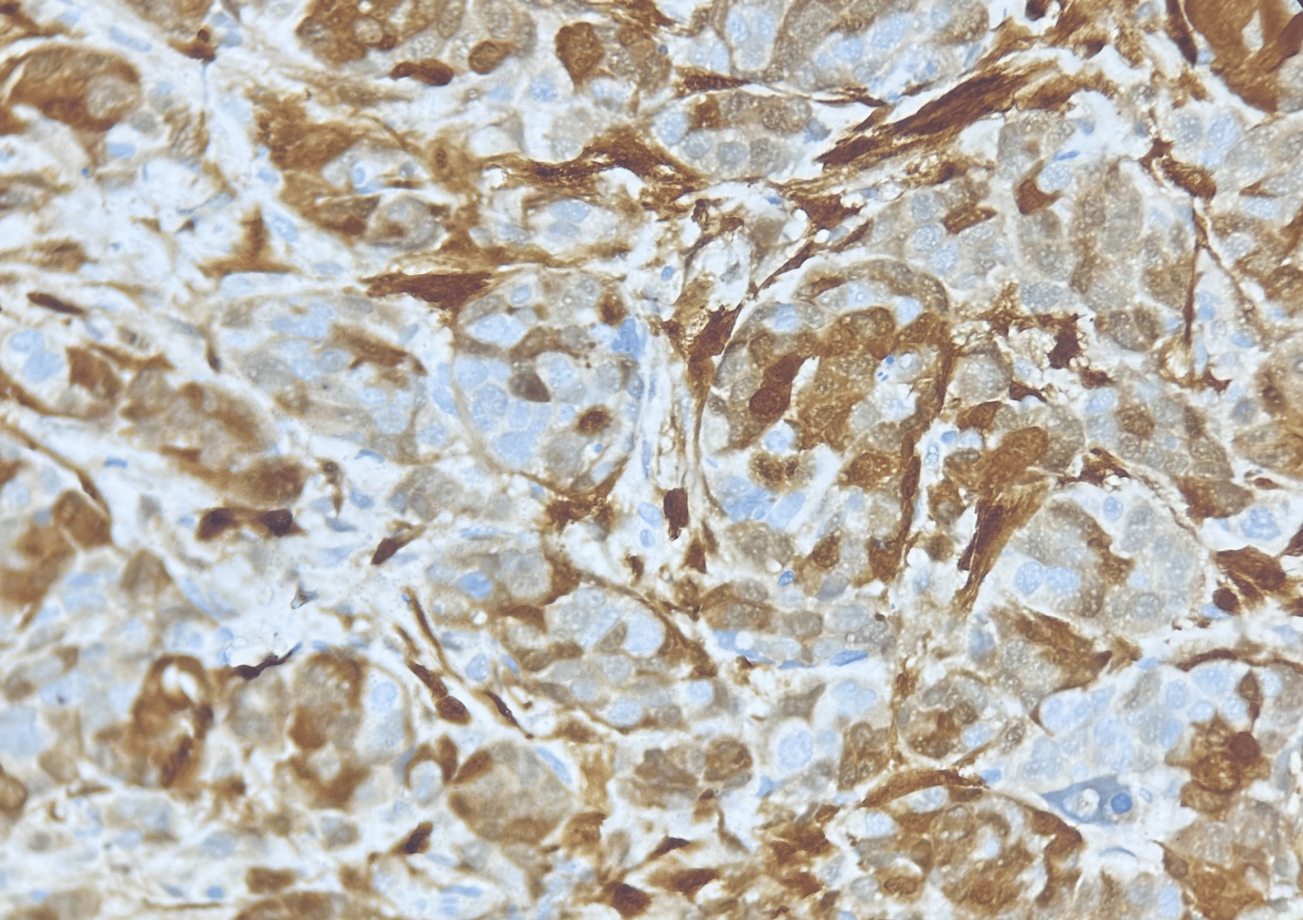
Immunohistochemistry showing nuclear positivity with WT1 (Wilms tumor 1) Magnification 40X.

Immunohistochemical markers were positive (Figure 4) for WT1 (Wilms tumor 1) and calretinin and negative for napsin, TTF-1 (thyroid transcription factor-1), p40, and PAX8 (paired-box gene 8).

These findings led to the confirmation of the epithelioid subtype of malignant mesothelioma upon histopathological examination of the parietal pleural biopsy specimen. This case represents a rare clinical scenario with a female patient lacking a history of asbestos exposure yet afflicted with both pleural and peritoneal mesothelioma. The complexities of the case underscore the need for a comprehensive investigation of pleural effusion cases to unveil underlying rare causes such as mesothelioma. In addition, an FDG-PET/CT scan revealed metabolically active diffused suitable pleural thickening; nodules in the right lung parenchyma; metabolically active peritoneal deposits; and mediastinal, retroperitoneal, axillary, and right supraclavicular lymphadenopathy.

Consequently, a diagnosis of synchronous pleural and peritoneal mesothelioma was established, indicating advanced stage IV mesothelioma. The chronicity of respiratory symptoms and constitutional symptoms such as anorexia and weight loss pointed toward either tubercular or malignant pleural effusion. Despite the exudative nature of pleural fluid analysis and lymphocyte predominance favoring a tubercular effusion, poor response to antitubercular therapy and borderline adenosine deaminase levels heightened suspicion of a malignant pathology, ultimately leading to the discovery of malignant pleural mesothelioma.

Chemotherapy was initiated with a combination of carboplatin and pemetrexed due to the staging findings indicating the inoperability of the tumor. The patient is currently undergoing chemotherapy with carboplatin 350 mg and pemetrexed 600 mg every three weeks, with two cycles completed to date. Regular follow-up visits are scheduled every three weeks for chemotherapy administration, and the patient is able to perform routine activities.

## Discussion

The reported worldwide cases of mesothelioma totaled approximately 30,870, with a standardized rate of 0.30 per 100,000 individuals [1]. Notably, the incidence among females was significantly lower than that among males [1]. Approximately 70% of pleural mesothelioma cases are associated with proven asbestos exposure, including instances of exposure through household contact with workers [2]. Asbestos fibers have the potential to accumulate in the lung tissue and traverse the visceral pleura, ultimately leading to the formation of malignant mesothelial plaques on the pleural surface [5]. Although the pathophysiology of peritoneal mesothelioma remains unclear, some theories propose that asbestos fibers originating from the lungs may migrate to the peritoneum through the lymphatic system [5]. Notably, our female patient under consideration had no documented history of asbestos exposure, either occupationally or environmentally. Additional risk factors include heterozygous germline BAP1 pathogenic mutations, exposure to radiotherapy, ionizing radiation, and simian virus 40 (SV40) exposure [1].

Malignant pleural mesothelioma typically manifests radiologically as asymmetric nodular pleural thickening, unilateral pleural effusion, atelectasis, encased lung, and lung volume loss. As a result, chest X-rays are commonly used as an initial screening tool to detect potential malignant pleural mesothelioma, prompting further imaging examinations if suspicious findings are identified [3]. CT scans represent the primary investigative modality. During staging, CT scans are critical for assessing the local spread of the disease from the pleural space, particularly into the chest wall, diaphragm, and mediastinum. Patients presenting with signs of transdiaphragmatic spread, involvement of multiple areas of the chest wall, invasion into vital structures within the mediastinum, presence of disease outside the thoracic cavity, or nodal involvement on the opposite side of the chest are typically not considered suitable candidates for surgery [3].

The 2021 update to the WHO classification of pleural tumors recognizes three primary histologic subtypes: epithelioid, sarcomatoid, and biphasic. This classification system offers practical diagnostic guidance by associating each mesothelioma subtype with specific potential differential diagnoses, thereby aiding the pathology workup process [6]. Calretinin plays a crucial role in identifying cells derived from mesothelial tissue, and strong staining for epithelial membrane antigens can effectively differentiate epithelioid mesothelioma from reactive hyperplasia and adenocarcinoma [2].

The use of FDG-PETCT in the management of malignant pleural mesothelioma is crucial for the identification of occult metastases, particularly those that may not be detectable on CT scans alone, such as extrathoracic and nodal metastases [3]. Malignant mesothelioma staging has been a subject of debate, and Rusch et al. developed a staging system from the International Mesothelioma Interest Group (IMIG) to provide precise anatomical definitions of the primary tumor's local extent [7]. This system serves as a foundation for analyzing future clinical trials of emerging treatment strategies.

In our specific case, a diagnosis of stage IV malignant mesothelioma was established, and treatment commenced with a chemotherapy regimen involving carboplatin and pemetrexed under the supervision of a medical oncologist. However, advancements in diagnostic methodologies have led to the identification of the condition at earlier stages, potentially contributing to an amplified lead-time bias in gauging survival outcomes. For such patients, prioritizing palliative strategies to recognize and manage physical and emotional symptoms is crucial [2].

In retrospect, our case presents several considerations. When pleural fluid analysis yields inconclusive results, seeking histopathological confirmation of the diagnosis rather than initiating empirical therapy, especially with antitubercular drugs, is advisable, considering the potential side effects of the drugs. Despite the challenges, our institution provided all investigations and treatments free of charge. Nonetheless, the significance of this case should not be underestimated, given the rarity of the disease. The complex diagnostic journey outlined in this case offers valuable educational insights and underscores the effectiveness of utilizing auxiliary examination methods in diagnosing mesothelioma.

This case highlights a step-by-step approach to identifying malignant pleural mesothelioma, beginning with the analysis of pleural fluid in the laboratory to discern its exudative properties. Subsequent imaging tests such as CT scans reveal anomalies in the pleura and peritoneum. Medical thoracoscopy enables the retrieval of pleural tissue for biopsy, and histopathological examination and immunohistochemistry are pivotal in conclusively confirming the diagnosis. Notably, the patient experienced significant relief from symptoms through intercostal tube-guided drainage of pleural fluid. We successfully confirmed the diagnosis within two weeks of admission and achieved clinical stability. The patient is now under regular follow-up and monitoring, and we are optimistic about achieving improved survival outcomes. Hence, we advocate for continued research into mesothelioma treatment with its tremendous potential for further advancements in the future.

## Conclusions

A systematic stepwise approach to pleural effusions is necessary for successful clinical outcomes. Malignant effusions should be retained as a differential diagnosis while investigating any exudative pleural effusions. The malignant mesothelioma presents itself as an infrequent neoplasm characterized by a dismal prognosis. Detecting it early and promptly commencing treatment can extend lifespan and enhance the well-being of individuals, particularly those of a younger age. The intricacies of our case emphasize the vital need for a comprehensive examination of cases involving pleural effusion in order to uncover rare underlying etiologies such as mesothelioma.

## References

[1] Huang J, Chan SC, Pang WS (2023). Global incidence, risk factors, and temporal trends of mesothelioma: a population-based study. J Thorac Oncol.

[2] Brims F (2021). Epidemiology and clinical aspects of malignant pleural mesothelioma. Cancers (Basel).

[3] Del Gobbo A, Fiori S, Gaudioso G (2014). Synchronous pleural and peritoneal malignant mesothelioma: a case report and review of literature. Int J Clin Exp Pathol.

[4] D'Agostino HP, Edens MA (2024). Physiology, pleural fluid. StatPearls [Internet].

[5] Rusch VW, Giroux D, Kennedy C (2012). Initial analysis of the international association for the study of lung cancer mesothelioma database. J Thorac Oncol.

[6] Borczuk AC, Cooper WA, Dacic S (2021). Tumors affecting the pleura and pericardium. Thoracic Tumours: WHO Classification of Tumours.

[7] Katz SI, Straus CM, Roshkovan L (2023). Considerations for imaging of malignant pleural mesothelioma: a consensus statement from the International Mesothelioma Interest Group. J Thorac Oncol.

